# Stuttering: A Disorder of Energy Supply to Neurons?

**DOI:** 10.3389/fnhum.2021.662204

**Published:** 2021-08-26

**Authors:** Per A. Alm

**Affiliations:** Department of Neuroscience, Uppsala University, Uppsala, Sweden

**Keywords:** stuttering, dopamine, basal ganglia, speech, metabolism, aerobic glycolysis, glycolysis, glucose

## Abstract

Stuttering is a disorder characterized by intermittent loss of volitional control of speech movements. This hypothesis and theory article focuses on the proposal that stuttering may be related to an impairment of the energy supply to neurons. Findings from electroencephalography (EEG), brain imaging, genetics, and biochemistry are reviewed: (1) Analyses of the EEG spectra at rest have repeatedly reported reduced power in the beta band, which is compatible with indications of reduced metabolism. (2) Studies of the absolute level of regional cerebral blood flow (rCBF) show conflicting findings, with two studies reporting reduced rCBF in the frontal lobe, and two studies, based on a different method, reporting no group differences. This contradiction has not yet been resolved. (3) The pattern of reduction in the studies reporting reduced rCBF corresponds to the regional pattern of the glycolytic index (GI; Vaishnavi et al., 2010). High regional GI indicates high reliance on non-oxidative metabolism, i.e., glycolysis. (4) Variants of the gene ARNT2 have been associated with stuttering. This gene is primarily expressed in the brain, with a pattern roughly corresponding to the pattern of regional GI. A central function of the ARNT2 protein is to act as one part of a sensor system indicating low levels of oxygen in brain tissue and to activate appropriate responses, including activation of glycolysis. (5) It has been established that genes related to the functions of the lysosomes are implicated in some cases of stuttering. It is possible that these gene variants result in a reduced peak rate of energy supply to neurons. (6) Lastly, there are indications of interactions between the metabolic system and the dopamine system: for example, it is known that acute hypoxia results in an elevated tonic level of dopamine in the synapses. Will mild chronic limitations of energy supply also result in elevated levels of dopamine? The indications of such interaction effects suggest that the metabolic theory of stuttering should be explored in parallel with the exploration of the dopaminergic theory.

## Introduction

Stuttering is a disorder of speech. The core symptoms manifest as an intermittent loss of volitional control of speech movements, resulting in various forms of speech disruptions, commonly described as repetitions, prolongations, or blocks (Perkins, 1990; Bloodstein and Bernstein-Ratner, 2008). Research on the neurological underpinnings of stuttering has made remarkable progress over the last two decades; yet, our understanding of the nature and mechanisms of the disorder can still be described as fragmentary, with a range of proposed theories.

The present hypotheses and theory article was written as part of a series of articles aiming to analyze and integrate research findings on stuttering to date. The first article in the series focused on the role of streptococcal infections as a cause of stuttering (Alm, 2020). The second article was a more general review of the anatomy and functions of the dopamine system, and the mechanisms for the automatization of movement sequences, in particular in relation to speech and stuttering (Alm, unpublished manuscript). The aim of the present article is to review and discuss indications that stuttering is related to the supply of energy to neurons. The article begins with a brief review of the energy metabolism in the brain, followed by a discussion of the findings and observations related to stuttering and cerebral metabolism, primarily considering the electroencephalography (EEG) power spectrum at rest, brain imaging of the cerebral blood flow at rest, genetics, and biochemical measures.

### Energy Metabolism in the Brain

#### A Debated Topic

The brain does not move and it represents only about 2% of the human body mass, but it still consumes about 20% of the total body energy budget (Herculano-Houzel, 2012). The major energetic burden in the brain comes from the firing of synapses (Magistretti and Allaman, 2015). The last decades have seen a changing and more complex picture emerge of the energy metabolism of the brain (e.g., Vaishnavi et al., 2010; Rogatzki et al., 2015; Schurr, 2018; Yellen, 2018; Calì et al., 2019; Barros et al., 2021). This has opened new perspectives regarding the possible causal mechanisms of various neurological and psychiatric disorders. Though it is outside the scope of the present article to discuss these mechanisms in-depth, a brief overview will be attempted here^1^.

#### Glycolysis

In the “classical model,” there are two main pathways for glucose metabolism: an aerobic pathway, involving the mitochondria, and an anaerobic pathway, resulting in lactate as a (supposedly) harmful waste product. Both pathways produce adenosine triphosphate molecules (ATP) as fuel for the cells, though the aerobic process is 19 times more effective for ATP production than the anaerobic process. Therefore, the anaerobic pathway has been considered to only have the function of providing emergency support when the supply of oxygen is insufficient. The first step in both pathways is the splitting of the glucose molecule, i.e., *glycolysis*. The term glycolysis has often been associated with *anaerobic glycolysis*, resulting in lactate, but glycolysis is the first step also in the aerobic metabolism of glucose. More recently it has been claimed that lactate is always the result of glycolysis, and that lactate is the mitochondrial oxidative substrate (Rogatzki et al., 2015; Schurr, 2018).

#### Non-oxidative Glycolysis With Oxygen Available: “Aerobic Glycolysis”

In a seminal study, Fox et al. (1988) reported that activation of the primary visual cortex resulted in about a 50% increase in regional cerebral blood flow (rCBF) and glucose uptake, but only a 5% increase in oxygen uptake. Similarly, Madsen et al. (1995) found that a cognitive task resulted in a global increase in glucose uptake of 12% without a change in the oxygen uptake. These data suggested that during a momentary need for energy the brain uses non-oxidative metabolism of glucose. Non-oxidative glycolysis is much faster than the oxidative production of ATP, making it a good “first-responder” to acute energy needs of the neurons even when oxygen is available (Díaz-García and Yellen, 2019). The term “aerobic glycolysis” has become established for this phenomenon, i.e., utilization of non-oxidative glycolysis in the presence of oxygen (Pellerin and Magistretti, 1994; Vaishnavi et al., 2010; Yellen, 2018). The term was adopted from research on the metabolism of cancer cells, which tend to show this characteristic (Lunt and Vander Heiden, 2011).

#### Lactate as a Fuel for Neurons

Another seminal paper in this context was published by Schurr et al. (1988), who found that synaptic firing in hippocampal slice preparation was supported by lactate, without glucose. An influential theory in line with this result is the astrocyte-neuron lactate shuttle (ANLS) model, see Calì et al. (2019) and Barros et al. (2021) for recent updates. In brief, based on Calì et al. (2019), it is proposed that neurons consume glucose (diffusing from the capillaries) and lactate (produced by the astrocytes and released in proximity to the synapses). The astrocytes are assumed to use both glucose and stored glycogen for the production of lactate. According to this model, lactate is the preferred substrate for neuronal energy during periods of intense neuronal firing. In addition, it has been proposed that the release of lactate from the astrocytes signals long-term potentiation (LTP), implicating lactate in learning (Calì et al., 2019; Descalzi et al., 2019). The only storage of glucose in the brain is in the form of glycogen, primarily in the astrocytes (Bak et al., 2018; DiNuzzo, 2019). Recent research has come to emphasize the importance of glycogen in the brain, e.g., that “any interference with normal glycogen metabolism in the brain severely affects neuronal excitability and disrupts memory formation” (DiNuzzo, 2019, p. 1455).

### Variations of “Aerobic Glycolysis” by Brain Regions and Age

**Figure 1:**
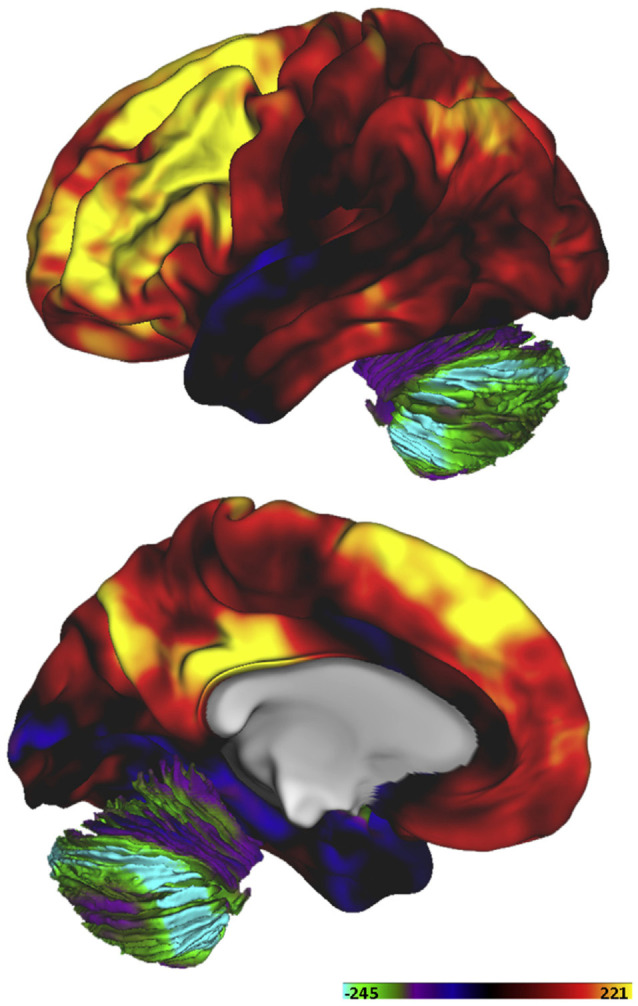
Variations in the utilization of aerobic glycolysis in the human brain of healthy adults expressed as a “glycolytic index (GI)”. Reprinted from Goyal et al. (2014), with permission.

Interestingly, it has been claimed that the metabolic properties of neuronal tissue differ substantially between different regions of the brain, and by age. By means of positron emission tomography (PET), regional and age-related differences in “aerobic glycolysis” were quantified by Raichle’s group, using a combination of tracers (Vaishnavi et al., 2010). With this method, they calculated a *glycolytic index* (GI), representing regional variations in metabolism. Basically, regions with a high GI normally show a high consumption of glucose in relation to the consumption of oxygen, interpreted as a high level of glycolysis and production of lactate. The resulting map is shown in Figure 1. The map shows that, in particular, regions within the frontal lobe show high levels of glycolysis, with lower levels in the posterior half of the brain, especially in the cerebellum and the medial temporal lobe. The regions with the highest GI are assumed to use glycolysis for about 25% of their energy consumption at rest, while the cerebellum only uses about 2%. The highest GI is in the inferior frontal gyrus, with a GI from 116 to 142, and the dorsolateral prefrontal cortex, with a GI of 120. This can be compared with a GI of approximately −80 in the hippocampal formation. The striatum shows a relatively high GI, approximately 70 (Vaishnavi et al., 2010).

**Figure 2:**
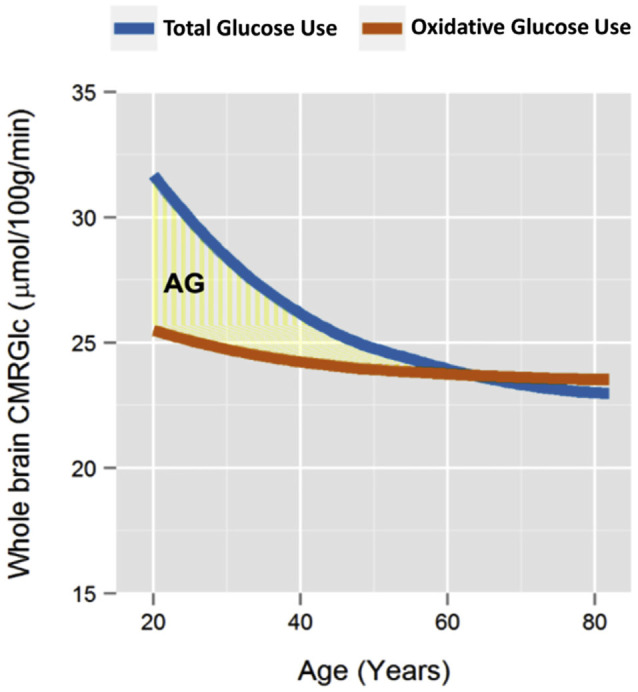
Results of a meta-analysis of whole-brain metabolism in relation to age, in cognitively normal adults. The blue line shows the total use of glucose, while the brown line shows the use of glucose with oxidative metabolism. The light-yellow area shows the amount of glucose that was metabolized by glycolysis, despite oxygen being available, i.e., “aerobic glycolysis”. Reprinted from Goyal et al. (2017), with permission. The blue line being lower than the brown line after the age of 60 is likely an artifact from fitting the trend lines.

The cerebral glycolysis varies strongly with age. In newborns, more than 30% of the brain glucose is metabolized by glycolysis (Vaishnavi et al., 2010). Figure 2 shows the changes in adults for the cerebral metabolism at rest (Goyal et al., 2017). At 20 years of age, about 20% of the glucose metabolism at rest is by glycolysis, which is reduced to zero at about 60 years of age, in cognitively normal persons. This age dependence has been discussed in terms of the specific role of cerebral lactate in neuronal plasticity and learning (Goyal et al., 2017; Descalzi et al., 2019). A temporary increase in glycolysis has been observed in cortical regions involved in motor learning, for hours after the training of a motor task (Shannon et al., 2016). This observation suggests that GI is not static, but at least partly a dynamic measure, reflecting the momentary level of plastic changes to the synaptic network. With increasing age, the number of new experiences and behaviors tends to go down. It would be of interest to compare the effect of novel motor training on glycolysis at different ages—would increased glycolysis be shown also at old age after novel motor training?

## Reduction of Beta EEG Power at Rest Related to Stuttering

### Effects of Hypoxia and Hyperventilation on the EEG Power Spectrum

One method to get information about the metabolic state of the brain is to analyze the power spectra obtained from electroencephalogram (EEG), as hypoxia and other disturbances affect the pattern of frequencies in the EEG power spectrum. Experimentally, there have been several attempts to model low-degree hypoxia in healthy humans, such as using a low-pressure chamber (Kraaier et al., 1988), reduced oxygen content of the air (Van der Worp et al., 1991), and pharmacologically induced cerebral vasoconstriction (Kraaier et al., 1992). For example, the study by Kraaier et al. (1992) reported that the pharmacologically induced vasoconstriction resulted in an about 40% reduction of the CBF accompanied by reduction of the EEG power from 9 Hz and higher, but without the formation of abnormal levels of lactate. Overall, hypoxia tends to result in a relative slowing of the EEG frequencies, though with different effects on specific frequency bands.

Hyperventilation is an established method to provoke abnormalities of the EEG for diagnostic purposes. Hyperventilation has complex effects, resulting in a reduced level of carbon dioxide, i.e., hypocapnia, and an increased level of oxygen in the blood. Hypocapnia has a strong vasoconstrictive effect in the brain, such that hyperventilation can reduce the CBF by up to 60% (Kraaier et al., 1992). Hyperventilation also affects the EEG power spectrum. Similarly to experimental hypoxia, the general tendency is a slowing of the oscillations, with an increase in the power of slow waves, in particular those from 2 Hz and lower, and a relative decrease in the alpha and beta waves (Kraaier et al., 1992; Achenbach-Ng et al., 1994; Zwiener et al., 1998). An interesting observation from studies with various methods is that large reductions in CBF may occur with relatively limited acute mental effects.

### The EEG Power Spectrum at Rest in Persons Who Stutter

In the literature, the present author found three reports of EEG power spectra obtained at rest in adults who stutter (Finitzo et al., 1991; Joos et al., 2014; Saltuklaroglu et al., 2017)^2^, and one in children, including hyperventilation (Ozge et al., 2004). The main common finding was that there was a decrease in the beta band power (about 13–20 Hz) in the stuttering groups relative to controls. The reports also show a tendency towards a decrease in alpha band power and an increase in the slow delta band. Thus, the EEG power spectra at rest for people who stutter resemble the spectra observed during mild hypoxia or hyperventilation. The studies will be reviewed in more detail below.

#### Study of *Children* Who Stutter, EEG With Hyperventilation

Ozge et al. (2004) studied 26 children who stuttered, aged 3–12 years old, and a group of matched controls. The average EEG power spectrum of the stuttering children at rest can be described as a shift from faster to slower activity compared with control, with a 30% increase in delta (0.5–3 Hz), 23% reduction in alpha (8.5–12 Hz), and 15% reduction in beta (12.5–30 Hz) power. Interestingly, with hyperventilation, the EEG spectrum of the control group approached the average spectrum of the stuttering group at rest. For the stuttering children, hyperventilation primarily resulted in further reduction of the beta power. Interestingly, the within-group variation in beta power in the stuttering group was strikingly smaller compared with the variation within the control group. For the measurement at rest, the beta power standard deviation (SD) of the stuttering group was only 50% of the SD of the control group, and with hyperventilation, it was only 40% of the SD for the controls. This implies that the stuttering group was much more homogenous than the control group with regard to the beta power. This high homogeneity, in turn, may suggest that this group difference reflects a core aspect of stuttering, shared by most persons who stutter.

#### Studies of the EEG Power Spectrum in *Adults* Who Stutter

**Figure 3:**
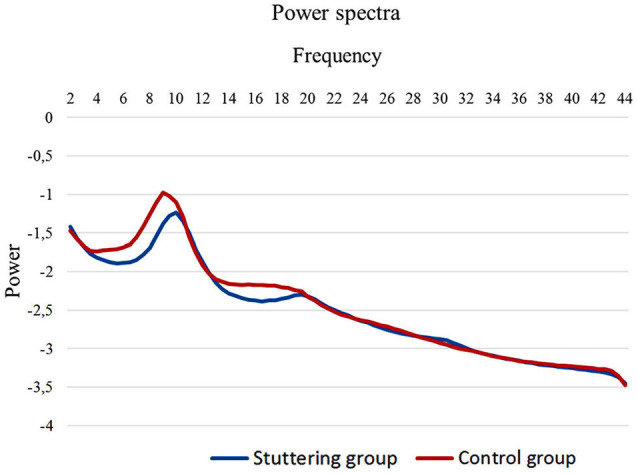
Mean whole-head electroencephalography (EEG) power spectra from adults who stutter and matched controls. Reprinted from Joos et al. (2014) according to Creative Commons license CC-BY 4.0.

Regarding studies of adults who stutter, Figure 3 shows the spectra obtained from one example of such a study, by Joos et al. (2014). The EEG was sampled by 19 channels with closed eyes for 5 min, with 11 participants in each group. After artifact rejection, the power EEG spectrum was calculated for all channels and averaged over all the electrodes and all the participants. There was significantly lower power in the theta (4–7.5 Hz) and beta 1 (12.5–18.5 Hz) bands for the stuttering group. The effect size of the group differences, as Cohen’s *d*, was 0.95 standard deviations for both bands. Inspection of the graph indicates that the peak alpha power was also lower in the stuttering group. Curiously, as in the study of children by Ozge et al. (2004), the stuttering group showed only about half the within-group variation as the control group for these frequency bands. The within-group variations in theta and beta power in the stuttering group were only 59% and 52% of the variations in the control group (variation measured as standard deviation).

The data from Saltuklaroglu et al. (2017) supports the finding of low beta power in adults who stutter, based on groups of 27 persons who stuttered and 27 controls, with a mean age of 27 years old. In addition, the graphs from Saltuklaroglu et al. (2017) showed a similar reduction in power of up to 30 Hz (no information available for frequencies above 30 Hz).

Last, also the study by Finitzo et al. (1991) supports the findings reported above, with the study finding significantly lower EEG power in the beta band, from 12.2 to 19.5 Hz. In fact, Finitzo et al. (1991) concluded:

*EEG spectral analysis shows a reduction in beta amplitude that is consistent with a mild reduction in absolute blood flow in these subjects (p. 251). A relationship between decreased Beta amplitude and reduced rCBF has long been recognized in ischemia … Beta activity is the first EEG frequency band to be affected in mild ischemia. Thus, quantitative Beta reduction in stutterers may have a correlate in rCBF hypoperfusion … Hypoperfusion can be due to autoregulatory phenomena at the neuronal level since blood flow is related to the activity and metabolic demands of the neurons involved (p. 257–258)*.

Their study is of special interest, as they also measured absolute rCBF in the same participants. These results are discussed in the next section.

#### Additional Comments

##### Beta Power and Cerebral Metabolism in ADHD

There are reports that reduced beta power at rest is a trait of attention deficit hyperactivity disorder (ADHD, e.g., the summary in Clarke et al., 2002), especially in the inattentive subtype (Buyck and Wiersema, 2014). However, this could not be confirmed by Arns et al. (2018). Medication with methylphenidate in ADHD tends to increase the beta power (Loo et al., 2016), showing a relation between the EEG power spectrum and the dopamine tone. In this context, it is of interest that ADHD has been proposed to be an “energy deficiency syndrome,” by Todd and Botteron (2001). Their proposal is in line with the present proposal on stuttering, though it has received little attention, with only 23 citations in PubMed. These authors hypothesized that dopamine affects the glycogen metabolism of the astrocytes, which, in turn, was assumed to affect the symptoms of ADHD. It is clear that a substantial subgroup of persons who stutter show traits of ADHD, in particular symptoms related to inattention (Alm, 2014; Druker et al., 2019; Tichenor et al., 2021).

##### Effect of Hyperventilation on Stuttering?

With regard to hyperventilation and stuttering, Johnson et al. (1959) investigated this topic, based on the reasoning that hyperventilation results in tetany, i.e., involuntary contractions of muscles. The participants hyperventilated until signs of tetany appeared, and performed readings of text with and without hyperventilation. However, no difference in the frequency of stuttering could be observed in relation to hyperventilation. The effects of hyperventilation are complex, making it difficult to interpret this report.

## Absolute Level of Cerebral Blood Flow in Stuttering

### Conflicting Data on CBF

#### Pool et al. (1991) vs. Ingham et al. (1996) and Braun et al. (1997)

The data in the literature on the absolute level of rCBF in stuttering are conflicting. Most brain imaging studies can not report the absolute level of rCBF, only relative changes, for methodological reasons. In parallel to the study of the EEG power spectra by Finitzo et al. (1991), reviewed above, Pool et al. (1991) studied the rCBF of the same participants. The method they used was [^133^Xe] SPECT during rest with open eyes. The results were presented for 22 regions of interest in one horizontal cross-section of the brain, from 20 adults who stuttered and 43 control persons. The authors claimed that the results showed there was a global reduction in the absolute CBF, on average approximately 20%. The combined finding of reduced CBF and reduction of EEG beta power in the same participants provided support for reduced metabolism.

The CBF study was questioned on several grounds, by Viswanath et al. (1992) and Fox et al. (1993), but was defended by the authors. A central argument in the critique was that a 20% global reduction of CBF would be expected to lead to more widespread effects, while the authors maintained that large variations in CBF may occur without obvious deficits. Another criticism was that at least a part of the control group was not acquired concurrently with the experimental group, which could make the results vulnerable to technical problems. The authors replied that they did run concurrent control persons, and when comparing these to the earlier sample, no significant differences were found (Fox et al., 1993). Yet another criticism concerned the specificity of the regions of interest (Fox et al., 1993), which, however, may not be of relevance when considering global differences in CBF.

Later, Ingham et al. (1996) and Braun et al. (1997) reported on the absolute CBF at rest of persons who stutter, based on [^15^O]-labeled water PET. They did *not* find support for any widespread group differences in absolute blood flow. The study by Ingham et al. (1996) included 10 persons who stuttered and 19 controls, with a mean age of 32 years old, whereas the study by Braun et al. (1997) included 18 persons who stutter and 20 controls, and the mean age was approximately 35 years old. PET with [^15^O]-labeled water is considered a reliable method for quantification of the absolute CBF (Carroll et al., 2002). Overall, the null results from the two later studies generally have been considered to settle the debate, that stuttering persons show no gross differences in absolute CBF.

**Figure 4:**
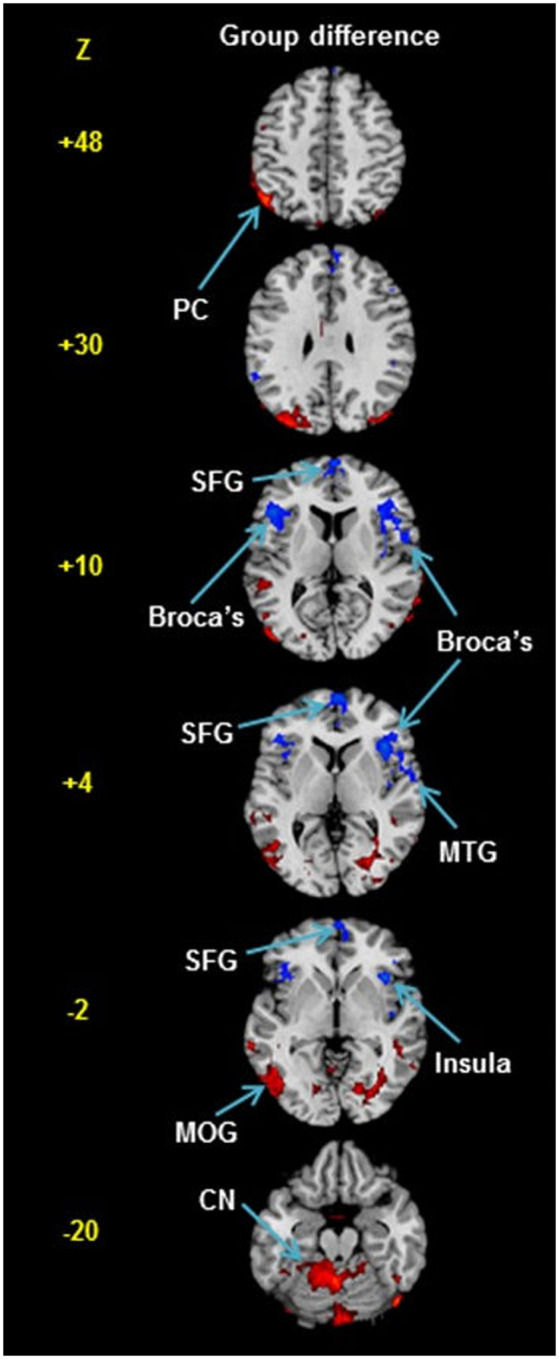
Group difference in rCBF from Desai et al. (2017). The rCBF was measured at rest using arterial spin labeling magnetic resonance imaging. The participants were 26 persons who stuttered and 36 typically fluent controls. Please note that the individual rCBF maps were normalized to *Z*-scores so that all participants showed the same mean and standard deviation. Blue and red voxels indicate regions with lower vs. higher relative rCBF in the stuttering group, with a threshold of *p* < 0.05, corrected for multiple comparisons. The normalization masks any global differences in rCBF. It is, therefore, possible that no regions showed higher absolute level of rCBF in the stuttering group, but instead a global reduction of absolute rCBF. The red regions are strongest in the cerebellum; overall, the pattern corresponds well with the map of regional differences in the glycolytic index shown in Figure 1. Abbreviations: PC, parietal cortex; SFG, superior frontal gyrus; MTG, middle temporal gyrus; MOG, middle occipital gyrus; CN, cerebellar nuclei. Reprinted from Desai et al. (2017) with permission.

However, a more recent study of rCBF in stuttering did actually report group differences in rCBF, at least partly in line with Pool et al. (1991). This was the study by Desai et al. (2017), who used arterial spin labeling magnetic resonance imaging (ASL MRI). This is a method with the capability to quantify absolute levels of rCBF; however, in this publication, only normalized results were reported: The rCBF data for each individual were normalized to a *Z*-score map, so that all individuals had the same mean and standard deviation, with the aim to highlight regional differences. The study involved 26 participants who stuttered, aged 5–51 years old, and 36 typically fluent controls. See group difference map in Figure 4.

Similar to Pool et al. (1991), reductions in rCBF were reported in the anterior half of the brain: in particular in the bilateral BA44 (= posterior Broca’s area) and the superior frontal gyrus (significance *p* < 0.05 after correction for multiple corrections, uncorrected *p* < 0.005). The severity of stuttering was correlated with the reduction in the left BA44, the Wernicke’s area and the left auditory cortex at rest. In the posterior half of the brain, on the contrary, relative *increases* were reported: in the left angular gyrus, middle occipital gyrus, and in the cerebellum. The normalization of the data masked any differences in the absolute rCBF. Normalized results will per definition show a balance between increases and decreases, as both groups will have the same mean. No other study suggests an elevated level of absolute rCBF in the posterior half of the brain in persons who stutter, suggesting that widespread increases are an unlikely result. Alternatively, if there are no regions with absolute increases in the stuttering group, it would imply that the global CBF is reduced in the stuttering group in Desai et al. (2017), similar to the result reported by Pool et al. (1991). To conclude, in case there are no regions with an absolute increase of rCBF in the stuttering group in Desai et al. (2017), the result appears to be very similar to the result in Pool et al. (1991) both in terms of reduced global CBF and the anterior-posterior gradient.

In summary, these four studies of rCBF at rest can be divided into two studies reporting no differences in absolute rCBF, based on [^15^O]-labeled water PET, and two studies reporting frontal reduction of rCBF. Can these contradictory results be reconciled? It seems clear that more information and data are needed to elucidate this issue.

### Correlation Between rCBF and the Regional Glycolytic Index

**Figure 5:**
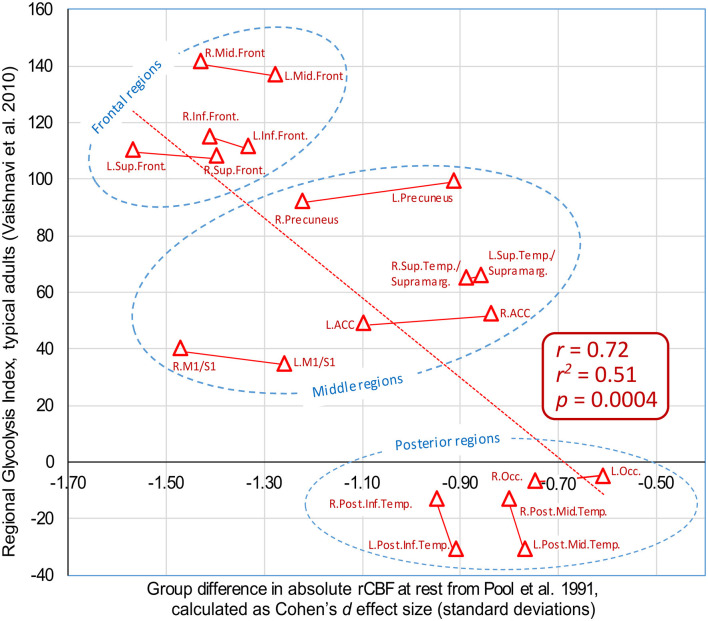
Plot of group differences in absolute regional cerebral blood flow (rCBF) as reported by Pool et al. (1991), vs. regional glycolytic index (GI) in healthy subjects, as reported by Vaishnavi et al. (2010). The group differences in rCBF are plotted as the effect size for each region, calculated as Cohen’s *d* [standard deviation (SDs)]. Positive GI values mean that the area has a high level of glycolysis, whereas negative GI values imply low levels of glycolysis. The approximate locations of the regions of interests (ROI) in Pool et al. (1991) were estimated by the present author. Data are available in the Supplementary Information. ACC: anterior cingulate cortex; Occ: Occipital cortex, approximately BA 17, 18, 19. M1/S1: precentral and postcentral gyri.

When the present author encountered the map of GI shown in Figure 1, the results from Pool et al. (1991) were recalled in memory, of reduced rCBF primarily in the frontal cortex regions. As the GI is based on measures of CBF, there might be a close physiological link. Naturally, the question arose: Is there a correlation? In order to estimate the correlation, the corresponding regions of GI from Vaishnavi et al. (2010) were approximated in relation to the region of interests (ROI) outlined in Pool et al. (1991). Thereafter, the effect sizes for the regional differences of rCBF in Pool et al. (1991) were calculated, as Cohen’s *d* values. Finally, the effect sizes of the rCBF differences were correlated with the regional GI values from Vaishnavi et al. (2010). The figures are available in the Supplementary Material. The Pearson correlation is *r* = 0.72, with *r^2^* = 0.51, *p* = 0.0004, see the plot in Figure 5. It is striking from the plot that the group differences reported by Pool et al. (1991) are not random: There is an anterior–posterior gradient, and the left/right homolog regions tend to appear together, in pairs. The eight regions with the largest reported reduction in rCBF are located from the central sulcus and into the frontal lobe.

A correlation between regional GI and reduction of rCBF in adults who stutter is further supported by results of Desai et al. (2017), as shown in Figure 4 (based on normalized data, giving both groups the same mean global CBF). The blue areas in Figure 4 indicates significantly lower relative rCBF in the stuttering group compared with controls (*p* < 0.05, corrected for multiple comparisons). All of these areas are located in regions with high regional GI, according to Figure 1. In contrast, the red areas in Figure 4 indicates regions with significantly higher relative rCBF in the stuttering group. All of these areas are located in regions with low regional GI, according to Figure 1.

In summary, the correlations between the reports of reduced rCBF in adults who stutter and the normal regional distribution of GI suggests a possible common underlying factor, involved both in stuttering and in cerebral aerobic glycolysis.

## Proton Chemical Shift Imaging of the Brain, at Rest

Magnetic imaging spectroscopy is a method for the non-invasive quantification of certain molecules, such as neurometabolites. In stuttering, one such study has been performed, by O’Neill et al. (2017), partly with the same participants as in Desai et al. (2017). The following molecules were measured: *N*-acetyl-aspartate plus *N*-acetyl-aspartyl-glutamate (NAA), choline compounds (Cho), and creatine (Cr). The results are not straightforward to interpret, but in summary, the authors concluded that: “Our investigation suggests that disturbances in neuronal or membrane metabolism contribute to the pathogenesis of stuttering” (p. E9).

Interestingly, magnetic imaging spectroscopy is also a method for the non-invasive measurement of cerebral levels of lactate. This method is used to study conditions with elevated levels of lactate; for example, related to impaired oxygenation (Hillary et al., 2007; Mitra et al., 2019) or suspected mitochondrial disorders (Kuang et al., 2018). Possibly, MRI spectroscopy of cerebral lactate during speech may be a method of interest for further studies of stuttering.

## Arnt2, a Gene for Initiation of Glycolysis in the Brain

### Regulation of Brain Metabolism

#### Mutation of the ARNT2 Gene Linked to Stuttering

As far as is known by the present author, the only genome-wide association study of stuttering published to date was performed by Kraft (2010). In genome-wide association studies, the relation between specific alleles and a trait is analyzed, while genome-wide linkage studies analyze the transmission of loci in families. The study by Kraft (2010) included 84 persons who stuttered and 107 matched controls. The fourth strongest association reported in the study was for the gene ARNT2, at the SNP rs11072922 (*p* = 0.000052, uncorrected for multiple comparisons). ARNT2 is of special interest in the current context as it is part of a mechanism for the cellular increase of glycolysis in the brain when the supply of oxygen is low. Variants at another location of this gene have previously been reported in autism (Chakrabarti et al., 2009; Di Napoli et al., 2015).

#### The ARNT2 Gene Is Involved in Sensing the Cerebral Oxygen Level, Activating Glycolysis

The ARNT2 gene encodes the ARNT2 protein. The ARNT2 protein acts as a partner for several other sensor proteins, responding to changes in the intracellular space. One of the key functions is that during hypoxia it binds with the hypoxia-inducible factor 1α (HIF-1α), and together they form the hypoxia-inducible factor 1 (HIF-1). The HIF-1, in turn, activates several genes which adapt the cell to the low-oxygen state, including an increase of glycolysis (Sharp et al., 2001; NCBI, 2021). HIF-1 has been described as a master regulator of oxygen homeostasis, with a central role in physiology, development, and pathophysiology (Semenza, 1999). At a normal level of oxygen, HIF-1α rapidly degrades, but low levels of oxygen inhibit the degradation so that it can bind to ARNT2 and form the HIF-1. More recently, it has been emphasized that the HIF-system for oxygen sensing is part of a complex system of hypoxia-sensing mechanisms, to sustain oxygen homeostasis. For a recent update, see SheikhBahaei (2020).

#### The Cerebral Expression of the ARNT2 Gene Roughly Matches the Glycolytic Index

Humans have two different genes which encode proteins that can act as subunits for HIF-1 to signal hypoxia: ARNT and ARNT2, which have both overlapping and unique functions (Sekine et al., 2006). ARNT is expressed richly in most body tissues, including the brain, while ARNT2 primarily is expressed in the brain (The Human Protein Atlas, 2021). In more detail, ARNT2 shows its strongest expression in the cerebral cortex and the basal ganglia, in neurons and glia, but shows low expression in the cerebellum, less than half of the cortical expression (The Human Protein Atlas, 2021). The ARNT gene shows the opposite pattern of expression in the brain, with the strongest expression in the cerebellum and lower in the rest of the brain. These patterns are of interest, as the expression of the ARNT2 gene corresponds to the pattern of the GI in the brain, with the lowest GI in the cerebellum, while ARNT shows the opposite distribution (Vaishnavi et al., 2010).

Interestingly, ARNT2 is weakly expressed in the dopaminergic nuclei (ventral tegmental area and substantia nigra pars compacta) compared to other brain regions (Dela Cruz et al., 2014). It should be noted that signaling low levels of oxygen is only one function of ARNT2, as it plays a role as a subunit for several different sensor proteins (NCBI, 2021).

#### Summary, ARNT2

In summary, the following observations link ARNT2 to the mechanism of “aerobic glycolysis” in the brain: (1) ARNT2 is part of a sensor mechanism for low levels of oxygen; (2) ARNT2 is primarily expressed in the brain; (3) the distribution of ARNT2 within the brain follows the GI differences between the cerebellum and the cerebrum; and (4) in contrast, ARNT shows the opposite distribution within the brain, with the strongest expression in the cerebellum. These observations support the role of the ARNT2 gene in the central nervous system (Maltepe et al., 2000), especially within the cerebrum. The present author can find no information regarding the possible role of ARNT2 for the regulation of neuronal supply of energy during normal conditions, for example during local brain activation resulting in a low level of available oxygen.

### Increased Risk for Early Hypoxic Injuries, Related to ARNT2 Variations?

Another aspect of impaired regulation of cerebral metabolism in relation to hypoxia is the increased risk of early hypoxic injuries, pre- or perinatal. Smith et al. (2016) summarized and discussed research related to pre- and perinatal risk factors and their interaction with genetics. The review is focused on ADHD, but their discussion can have wide applications. In addition, traits of ADHD sometimes co-occur with stuttering (Alm, 2014; Druker et al., 2019; Tichenor et al., 2021).

In relation to this, Drayna et al. (1999) concluded, based on their own data:

*One possibility suggested by these data is that roughly half of all cases of stuttering are due to inherited causes, while the other half is due to poorly understood but nongenetic factors. This hypothesis is consistent with the view that persistent stuttering of nongenetic origin is largely a male disorder (p. 1474)*.

The background for this conclusion by Drayna et al. (1999) was the observation of a large difference in gender ratio between stuttering persons reporting stuttering relatives and sporadic cases, with larger dominance of males in the sporadic cases. Which non-genetic factor may result in stuttering primarily in boys? In Alm and Risberg (2007) it was proposed that the main factor, in particular affecting boys, might be pre- or perinatal hypoxia, interacting with genetics and hormonal factors. Clinical and experimental experience have shown that males are more sensitive than females to pre- and perinatal hypoxia (Hill and Fitch, 2012), as a result of testosterone increasing the negative effects of hypoxia (Hill et al., 2011), and X-chromosome-linked genetic inhibition of apoptosis (Hill and Fitch, 2012). Hypoxia is associated with increased release of dopamine, which may have neurotoxic effects and contribute to deleterious effects of ischemic-hypoxia (Akiyama et al., 1991a; Davis et al., 2002; Yang et al., 2007). Laplante et al. (2012) reported opposite effects of perinatal hypoxia in male vs. female rats, on adult dopamine release in the nucleus accumbens. It has been proposed that the higher sensitivity to early hypoxia in males partially can account for the male preponderance in ADHD (Smith et al., 2016). Loss of dopaminergic neurons has not been indicated in these reports, but dysregulation of dopaminergic neurons and possible subtle striatal injuries have (Toft, 1999; Smith et al., 2016). Lou et al. (2004) reported a higher number of “empty” D2 receptors in adolescents with ADHD and a history of preterm birth and reduced neonatal CBF.

Overall, the available data provide support for an interaction effect between pre- and perinatal hypoxia and the male gender. As ARNT2 has an important role for the adaptation and protection of neurons during hypoxia, one would expect a tripartite interaction, among early hypoxia, male gender, and genetic variants affecting the function of ARNT2. It is here hypothesized that such interaction can account for part of the incidence of stuttering.

### High Blood Level of Nitric Oxide?

A study analyzing blood samples for indications of oxidative and nitrosative stress was published by Bilal et al. (2017), involving 40 children who stuttered, aged 3–17, and 40 children as the control group. The reported result was striking, with higher levels of all the reported compounds in the stuttering group. When calculating effect sizes^3^ (Cohen’s *d*) the following group differences were shown: nitric oxide* d* = 4.6 standard deviations; 3-nitrotyrosine *d* = 3.2; superoxide dismutase *d* = 2.5; malondialdehyde *d* = 2.4; and catalase *d* = 2.0. The report in the article indicates that there was no group overlap for nitric oxide.

These results are of great potential interest, but difficult to interpret. Primarily, there is a need to further investigate the results, to see if the group differences can be verified. The largest group difference was for nitric oxide (NO). Elevated NO can be a response to brain ischemia (Bolanos and Almeida, 1999). NO has a central and complex role in the cellular metabolism of the brain, including adaptation to hypoxia (Galkin et al., 2007; Man et al., 2014). The metabolism of energy can be affected by NO in multiple ways, such as an increase of the CBF (Toda et al., 2009) and inhibition of the transportation of oxygen into the mitochondria (Galkin et al., 2007). In relation to cerebral aerobic glycolysis, it is of special interest that NO has a great influence on the stability of the HIF-1α, and thereby on the activation or inhibition of glycolysis. NO has the ability both to activate glycolysis during the presence of oxygen and to inhibit glycolysis during hypoxia (Man et al., 2014). These functions make NO a factor of interest in the current context. However, there are also other mechanisms that can stabilize HIF-1α in the presence of oxygen (Semenza, 2010).

## Genes for Transportation of Enzymes to Lysosomes

### The Lysosomes and Intracellular Transportation

The best characterized genetic variants associated with stuttering relate to intracellular trafficking of molecules, which is an essential part of metabolism. Because of the established links to both stuttering and to cellular metabolism, these genetic findings need to be discussed in the current context.

These genes have been identified by the work of the Dennis Drayna group at the National Institute of Health, USA. The variants are genes related to the endosomal transport system, which has the function of sorting and transporting complex biomolecules to the lysosomes (Kulkarni and Maday, 2018). The lysosomes are known as the cellular stations for the degrading and recycling of material. Recent research indicates that the lysosomes have important control functions, sensing the availability of nutrients and influencing the metabolism of the cell (Xu and Ren, 2015; Lim and Zoncu, 2016; Lawrence and Zoncu, 2019). The lysosomes are cell organelles containing enzymes that are activated by the acid interior of the lysosome. The enzymes are hydrolases, which break down various biomolecules such as proteins and complex lipids and carbohydrates to their building blocks (Xu and Ren, 2015). These hydrolases are inactive as long as they are outside the lysosomes, because of the higher pH in the surrounding cytosol. The lysosomal hydrolases are synthesized outside the lysosomes together with a large number of other proteins. How can these hydrolases be selected and transported to the lysosomes, to do their work there? The main transportation pathway is through tagging with a mannose-6-phosphate group (M6P). Within the trans-Golgi network, an intracellular “sorting station,” the lysosomal hydrolases are marked with an M6P group, for transportation to the endosomes and further transportation to their final destination in the lysosomes (Coutinho et al., 2012).

### Four Genes Linked to Stuttering and Intracellular Transportation

In total, four genes involved in this transportation system have been linked to stuttering. Three of these genes encode enzymes that encode the M6P group, which has the function of being an address tag to the lysosomes. The three genes are GNPTAB, GNPTG, and NAGPA (Kang et al., 2010; Raza et al., 2016; Srikumari et al., 2020). In total, 81 different variants of these genes have been associated with stuttering (Raza et al., 2016). Impairments of the functions of these genes can be expected to impair the selection and transportation of lysosomal hydrolases to the lysosomes. A fourth gene linked to stuttering has also been characterized by the Drayna group: AP4E1 (Raza et al., 2015). AP4E1 is involved in the next step of transportation from the trans-Golgi network to the lysosomes (Raza et al., 2015). This implies that all four of these genes may be related to the same mechanism underlying stuttering, involving the transportation of enzymes to the lysosomes. In Frigerio-Domingues and Drayna (2017), the contribution of these genes to stuttering, in general, is discussed. They estimated that something like 12–20% of the total cases of stuttering can be explained by these gene variants.

Kang et al. (2010) presented the distribution of one of these variants—the GNPTAB Glu1200Lys mutation—in one Pakistani family. In this family, the mutation showed a gender difference in penetrance, with 100% of homozygotic males showing stuttering (9 out of 9), and 60% of homozygotic females (3 out of 5). For heterozygotic males, the penetrance was 79% compared to 25% for heterozygotic females. However, the level of penetrance has to be interpreted carefully, as it may well be combined with other genes increasing the risk for stuttering.

It should be noted that the carriers of these genes generally are in good health, not showing any other obvious symptoms than stuttering. This suggests that the effects of these gene variants are relatively mild and partial.

In summary, these results suggest that stuttering in some cases is related to a reduced amount of hydrolase enzymes in the lysosomes, resulting in a reduced rate of degradation of proteins and complex lipids and carbohydrates. There is a range of different lysosomal storage disorders, related to various aspects of physiology (Parkinson-Lawrence et al., 2010). Considering that these variants do not cause general health problems or widespread neurological dysfunction, it appears that the processing capacity of the lysosomes fulfills the baseline demand, but that the higher demands imposed by speech exceed the processing capacity of the cells. This may result in an accumulation of complex biomolecules in the cytoplasm and a shortage of recycled building blocks, such as amino acids, glucose, and lactate. In the current context, further analysis of the role of the lysosomes in cerebral aerobic glycolysis will be of importance.

Of particular interest is that the lysosomes play a role in glycogenolysis, i.e., the degradation of stored glycogen to glucose or lactate in the astrocytes, by the enzyme alpha-glucosidase (Adeva-Andany et al., 2016; Calì et al., 2019; Duran et al., 2019). As mentioned in the introduction, a well-functioning metabolism of glycogen has been shown to be essential for the brain (DiNuzzo, 2019). However, based on the discussion in Bak et al. (2018), it appears that the primary pathway for cerebral degradation of glycogen may be outside the lysosomes, by the enzyme glycogen phosphorylase. It is therefore possible that gene variants affecting the metabolic rate of the lysosomes do not have important effects on cerebral glycogenolysis.

### Mice With “Stuttering” Mutation in the GNPTAB Gene

#### Reduced Number of Vocalizations

In Barnes et al. (2016), mice were engineered to carry a homozygous mutation in the GNPTAB gene, corresponding to the Glu1200Lys mutation discussed above. These mice were in good health and behaved normally in a series of behavioral tests. The difference that could be detected was in the ultrasonic vocalization of pups. The pups normally produce bouts of vocalizations, separated by longer pauses. Overall, the pups with the mutation produced only 32% of the number of vocalizations per time unit, compared with the wild-type. The mean number of vocalizations per bout was somewhat smaller for the mice carrying the mutation, though not statistically significant (3.0 vs. 3.6). The main difference was a longer duration between the bouts.

#### Reduced Number of Astrocytes

Han et al. (2019) reported that two other mutations of the GNPTAB gene associated with stuttering resulted in the same reduction in vocalization as the mice discussed above. Immunohistochemistry showed a marked reduction in the number of astrocytes in these mice, in particular in the corpus callosum. The authors proposed that the results support hypotheses regarding deficits in intrahemispheric communication in stuttering. Further experiments suggested that only mice with a reduced number of astrocytes showed a decrease in vocalization. The finding that the strongest reduction of astrocytes in mice was localized to the corpus callosum fits with the expression pattern of the GNPTAB gene in the mouse brain, with the strongest expression in the corpus callosum (The Human Protein Atlas, 2021). However, this may differ in humans, as the human samples indicate the highest expression in the cerebral cortex and the basal ganglia, with relatively lower expression in the human corpus callosum (The Human Protein Atlas, 2021). In the human cerebral cortex, the GNPTAB gene is strongly expressed in neurons but at low levels in glial cells (astrocytes not specified, The Human Protein Atlas, 2021).

### Lysosomal Deficits May Affect Gray Matter Development

Chow et al. (2020) compared the expression patterns of two stuttering-related genes, GNPTG and NAGPA, with the pattern of magnitude of differences of gray matter volume between children with persistent stuttering and fluent controls. They reported a positive correlation, and that this pattern also correlated with the expression patterns of other genes involved in glycolysis and oxidative metabolism in the mitochondria. They discussed the possibility that impairment of lysosomal enzymes trafficking may lead to accumulation of damaged mitochondria and increased oxidative stress, with a negative effect on neurological development. It was also proposed that this may be related to the normal rapid increase in the cerebral metabolic rate occurring after 2 years of age, as shown by Chugani et al. (1987).

### Summary, Lysosomal Genes

This group of mutations is likely to result in a mild reduction of the lysosomal processing capacity, which, in turn, may result in a mild reduction of the cellular metabolic rate, with stuttering as the main symptom. This pathway is partly involved in the metabolism of glycogen stored in the astrocytes to glucose or lactate, as fuel for the neurons. Mice pups carrying this type of gene, associated with stuttering, show less frequent vocalization and a reduced number of astrocytes in the white matter. These findings support the hypothesis that a limitation in the supply of neuronal energy is an aspect of stuttering.

## Thiamine

Thiamine, also known as vitamin B_1_, is a compound necessary for the metabolism of carbohydrates and cellular energy supply, in particular for the central nervous system. It has been claimed that supplement of thiamine can reduce stuttering in some cases, which makes it relevant to discuss in this context.

Thiamine serves as a cofactor for several enzymes, mostly with mitochondrial localization (Dhir et al., 2019; Encyclopedia Britannica, 2021). Deficiencies in thiamine intake, or genetic disorders of transportation or metabolism of thiamine, can result in a wide range of disorders, often involving nervous tissues (McCandless, 2009; Marcé-Grau et al., 2019). More recently, developmental conditions such as autism spectrum disorder and delayed language development have been associated as possible effects of thiamine deficiency (Fattal-Valevski et al., 2009; Dhir et al., 2019). Some genetic defects of thiamine transport and metabolism result in specific neurological symptoms, such as degeneration of the striatum and generalized dystonia (Marcé-Grau et al., 2019).

There are two small studies reporting improvement of stuttering from thiamine supplement, in some persons who stutter. These results have to be described as uncertain, but their potential importance and their direct relation to the topic of this article makes them relevant for discussion. The first study was conducted by Hale (1951) with children who stuttered, age 2–8. The rationale for the study was to provide additional nutritional support for the development of the central nervous system, with additional energy and supply of carbohydrates, during the period of speech development. The study was double-blind with a cross-over design. The children received 30 mg of thiamine daily, or placebo, for 1 month, and thereafter the groups changed treatments for an additional month. The presentation of the results is poor by today’s standards; however, it was claimed that 80% of the children aged 2–3 years showed an observable improvement on thiamine, while little or no improvement could be observed in the children aged 5–8 years. A reflection that comes to mind is that the improvements reported at age 2–3 years may be due to the widespread spontaneous improvement of stuttering at this age (Yairi, 2004). The cross-over design may have made it possible to control for this, but the data were not reported with this level of detail. It was reported that if stuttering was markedly improved, it typically was improved within the first 2 weeks of thiamine treatment.

To the knowledge of the present author, no other controlled studies of thiamine for children who stutter have been reported. However, Schwartz (2011) reported that uncontrolled attempts to treat stuttering at age 2–4 with 30 mg of thiamine daily resulted in a dramatic reduction of stuttering in almost 60% of the cases, within 2 weeks. Cases showing no improvement after 2 weeks were unlikely to show later improvement following longer treatment.

In addition, Schwartz (2011) reported a preliminary randomized double-blind study of thiamine for adults who stutter. In the study, 19 adults who stuttered were randomly assigned to a treatment group and 19 to a placebo group. The treatment consisted of 300 mg of thiamine daily for 2 weeks. It was reported that six out 19 in the treatment group showed a “switch effect” with a dramatic improvement of their stuttering, from 9.1% stuttered syllables before treatment to less than 1% at the end of the treatment. The rest of the participants showed no significant improvement. An informal follow-up on these six cases for 5 years indicated that the improvement remained, as long as the thiamine supplement was continued (Schwartz, 2015).

The study of adults by Schwartz (2011) was replicated by Hum et al. (2017), though with 100 mg of thiamine daily instead of 300 mg, with 19 adults for 2 weeks. This study did not show a treatment effect of thiamine. The possibility cannot be excluded that the difference in dosage affected the result, as anecdotal reports claim a dosage effect, with 100 mg being inefficient compared with 300 mg (Kehoe, 2013).

The safety of high dosage thiamine supplementation is discussed in a report by the Committee on Toxicity (2003), part of the Food Standards Agency, UK. It concluded that there are insufficient data to establish a safe upper level for thiamine, though “the oral toxicity of thiamin and thiamin derivatives in humans is generally considered to be very low. No specific toxic effects of thiamin ingestion by humans have been identified” (p. 79). As an example, the Committee reported that doses at 5,000 mg daily and higher may cause reversible symptoms of headache, nausea, and insomnia. For nutrition therapy in adults, Sriram et al. (2012) recommend 100 mg three times a day when at risk for deficiency, and 200 mg three times a day for high suspicion or proven deficiency.

## Discussion

### Overview of the Results From the Review

#### EEG-Spectra at Rest Indicate Reduced Cerebral Metabolism

Four studies of the EEG power spectra at rest in persons who stutter were found in this review, one with children and three with adults. The main converging result was that all studies showed lower beta power compared with controls, and the overall tendency appeared to be a reduction of EEG power from the alpha band and higher, and some increase of power in the delta band (low frequency). Reduced beta power is considered a main characteristic of conditions involving limitations in the supply of neuronal energy. Possibly the most remarkable result was that in the two studies of which the within-group variation is available (Ozge et al., 2004; Joos et al., 2014), the stuttering groups showed about half the standard deviation for beta power compared with the control groups. In other words, the stuttering groups showed a surprising homogeneity in this respect, suggesting that it might reflect a core trait of stuttering.

#### Conflicting Reports on Absolute rCBF, Correlation With Glycolytic Index

The review on absolute rCBF resulted in two studies reporting significantly lower frontal lobe rCBF in the stuttering group (Pool et al., 1991; Desai et al., 2017), and two studies reporting no group differences in absolute levels (Ingham et al., 1996; Braun et al., 1997). An interesting aspect is that both of the two studies reporting no differences used [^15^O]-labeled water PET, while the studies reporting differences used other methods. It appears important to investigate this issue further. Is there any fundamental difference between the methods that caused the different results?

It is interesting to note that the studies by Pool et al. (1991) and Desai et al. (2017) reported a similar pattern of rCBF in the stuttering group, with an anterior-posterior gradient: low in the frontal lobe and relatively higher in the posterior half of the brain. As the results in Desai et al. (2017) only were presented as normalized rCBF, it is possible that the absolute CBF in Desai et al. (2017) matched the results of Pool et al. (1991) in terms of reduced global CBF.

Further, the rCBF patterns in Pool et al. (1991) and Desai et al. (2017) correspond to the regional variations in GI, as calculated by Vaishnavi et al. (2010). Regions with high GI show high consumption of glucose in relation to the consumption of oxygen, interpreted as high reliance on glycolysis with the production of lactate. The reductions of rCBF in the stuttering group in Pool et al. (1991) showed a correlation of *r* = 0.72 with the regional GI (*p* = 0.0004). This suggests that those regions with high reliance on glycolysis and production of lactate were the regions that showed the largest reduction of rCBF in the stuttering group in the studies by Pool et al. (1991) and Desai et al. (2017). Based on these results, it is here preliminary hypothesized that stuttering groups tend to show impairment of the cellular mechanism activating glycolysis and production of lactate during high demands for energy.

#### Proton Chemical Shift Imaging of the Brain, at Rest

One study of brain metabolites in persons who stutter was reviewed, a study based on magnetic imaging spectroscopy of the brain (O’Neill et al., 2017). The result of this study is difficult to interpret, but the authors claim that it indicates disturbances in neuronal or membrane metabolism.

#### ARNT2, a Gene for Initiation of Glycolysis in the Brain

ARNT2 was the gene that showed the fourth-strongest association with stuttering in the genome-wide association study by Kraft (2010). A main function of the gene is to produce a protein that is one part of a sensor mechanism (HIF-1) that detects low levels of oxygen in the brain and activates non-oxidative metabolism with glycolysis and production of lactate. ARNT2 is expressed in the cerebral cortex and the basal ganglia, with lower levels in the cerebellum. In the cerebral cortex, ARNT2 is expressed both in neurons and in glial cells. The function and the pattern of distribution imply that ARNT2 is a possible element in the mechanism underlying “aerobic glycolysis”.

A dysfunction of the ARNT2 genes would be expected to result in impaired adaptation to low levels of oxygen in the brain, for example by reduced activation of glycolysis and maybe also by the insufficient increase of the cerebral blood flow. This can be consistent with the reduction of EEG beta power in the stuttering group, and the reduction of frontal rCBF reported by Pool et al. (1991) and Desai et al. (2017). Furthermore, a dysfunction of ARNT2 would be expected to result in elevated sensitivity to pre- or perinatal hypoxia, in particular in boys. The interaction between early hypoxia and male gender is proposed to account in part for the unexplained non-genetic causation of stuttering in particular affecting males, which was discussed by Drayna et al. (1999).

In addition, one study of nitric oxide in the blood of children who stuttered, by Bilal et al. (2017), may be of great relevance in this context. The reported result is very striking, but the method of analysis appears to be relatively complicated, which implies that it needs to be confirmed before conclusions and hypotheses can be built on it. The blood level of nitric oxide was reported to be higher in the stuttering group, without overlap with the control group. Nitric oxide has a direct influence on the stability of HIF-1, and is able to both activate and inhibit glycolysis, during different conditions of oxygenation.

#### Genetic Impairment of Transportation of Lysosomal Enzymes

Four genes involved in the transportation of lysosomal enzymes to the lysosomes have been associated with stuttering by the Dennis Drayna group (Kang et al., 2010; Raza et al., 2016). The carriers of these genes are generally healthy except for stuttering. In mice, these mutations result in a longer delay between vocalizations than normal and a reduced number of astrocytes. It can be expected that these gene variants result in a decreased amount of degradation enzymes in the lysosomes, which may lead to the accumulation of complex biomolecules in the cytoplasm and a shortage of recycled building blocks. Both a reduced number of astrocytes and a reduced metabolic rate of the lysosomes may result in a reduced supply of energy to the neurons.

#### Thiamine Supports Oxidative Metabolism of Glucose

Thiamine has an essential role in the oxidative metabolism of glucose in the mitochondria. There are no published data indicating deficiency of thiamine in persons who stutter, but there are reports from preliminary studies suggesting that supplementation of thiamine may have an effect on stuttering in some persons who stutter. According to the reports, if the supplement is effective, the effect tends to show within 2 weeks. There are also anecdotal indications of a dose–response relationship. The available data need to be tested by larger systematic studies. It may be speculated that a supranormal level of thiamine can provide compensatory energy to the neurons in some cases of stuttering.

### Possible Causal Mechanisms, From Limitations of Energy Metabolism to Stuttering?

The above review has highlighted research findings that are compatible with the hypothesis that stuttering is related to some impairment of the energy supply to neurons. If it is assumed that this hypothesis is correct, in what way might a reduced supply of energy result in stuttering? Several mechanisms may be conceivable, but I will here discuss two proposals: (1) Speech stands out in relation to other behaviors in terms of the energy required by certain neurons. This could result in a reduced rate of firing by these neurons, causing stuttering. (2) The effect is indirect, for example, a reduced supply of energy causes an elevated tonic level of synaptic dopamine, which, in turn, results in stuttering.

#### Proposal 1: Insufficient Supply of Energy to Sustain Firing for Speech?

##### Persons Who Stutter Show Reduced Pre-speech Firing in the Motor System

**Figure 6:**
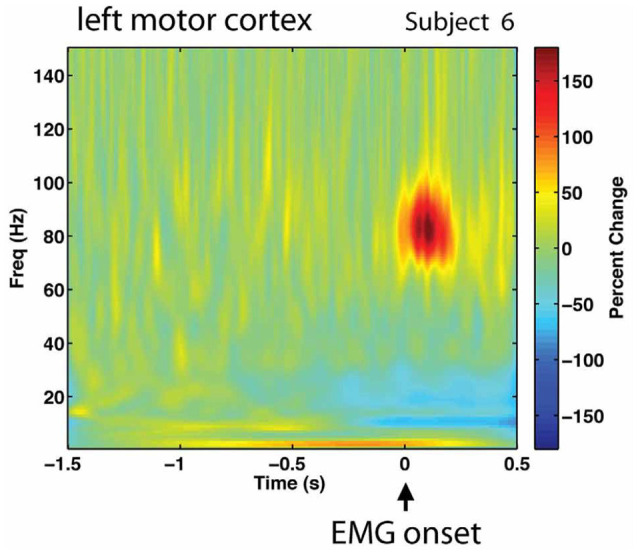
Gamma band activation, around 80 Hz, in the left primary motor cortex during right-s hand finger abduction. Signal recorded with magnetencephalography (MEG). Reprinted from Cheyne and Ferrari (2013) with permission.

The study by Neef et al. (2015) supports that reduced firing frequency of the motor system may be an important aspect of stuttering. By means of an experiment with transcranial magnetic stimulation (TMS), it was shown that persons who stuttered had a reduced level of activation of the primary motor cortex region of the tongue immediately before attempting to articulate a speech sound involving the tongue, compared with controls. In addition, the reduction of activity was correlated with the individual severity of the stuttering. It can be noted that the stuttering persons in Neef et al. (2015) were fluent in the experimental task, and still showed reduced premovement activation in the primary motor cortex. Normally, before a voluntary movement is initiated, there is a gradual increase of firing in the motor cortices and the putamen that can be detected hundreds of milliseconds to seconds prior to the movement (Romo and Schultz, 1992; Schultz and Romo, 1992). Figure 6 illustrates the high-frequency neuronal firing that is required within the primary motor cortex in relation to a finger movement. The red spot shows the firing at around 80 Hz, gamma band, during the movement.

##### Prolonged Pauses of Vocalization in Mice With a Gene Associated With Stuttering

As discussed above, the mice carrying the gene variant associated with stuttering showed prolonged pauses between bouts of vocalization (Barnes et al., 2016). It appears plausible that this effect could be the result of a reduced peak rate of energy supply to the motor system.

##### Are Cerebral Regions With a High Glycolytic Index (GI) the Most Affected in Stuttering?

If stuttering is related to an impairment of aerobic glycolysis, the greatest functional problems may be expected in regions with the highest glycolytic index (GI). This includes core regions for speech production, such as Broca’s area and the supplemental motor area (SMA); see Figure 1.

##### Insufficient Supply of Energy for Dopamine Neurons?

Bolam and Pissadaki (2012) argued that the sensorimotor dopamine neurons of the *substantia nigra pars compacta* (SNc) have extreme energy demands, as a result of the unusually high number of synapses in each neuron. They estimated that a single SNc dopamine neuron gives rise to between 1 million and 2.4 million synapses, and has a total axonal length of about 4.5 m. Some dopamine neurons in the substantia nigra pars compacta (SNc) appear to signal at the initiation of every single movement (Jin and Costa, 2015), which would require a sustained high level of energy supply during speech. On the other hand, dopamine neurons fire at a relatively low frequency, about >15 Hz during burst firing (Douma and de Kloet, 2020). Further, according to recent models of cerebral metabolism, e.g., by Magistretti and Allaman (2015), energy is supplied directly to the synapses by astrocytes. This would imply that the number of synapses per neuron may be of less importance for the energy load of the neurons. In summary, it is not clear that dopaminergic neurons are the most vulnerable to metabolic limitations during speech.

##### Speech Is a Complex, Sequential Motor Task

Speech is a motor process, characterized by being extended over time, with a sequence of varied complex motor actions and a high degree of automatization of the subunits. Most other activities that are extended over time tend to be repetitive and relatively simple, such as walking or running. Many other human movements are brief, such as picking up something. These characteristics of speech might make it particularly vulnerable to metabolic limitations. In addition, persons who stutter often produce single syllables or single words fluently, while connected speech imposes a higher frequency of stuttering (Bloodstein and Bernstein-Ratner, 2008). This could be a result of energy deficiency. However, it is also the case that stuttering often occurs in the initial position in utterances (Bloodstein and Bernstein-Ratner, 2008). This contradicts the hypothesis that metabolic limitations have a direct effect on speech production in persons who stutter. Similarly, persons who stutter typically are able to sing for extended periods of time without problems. Further, persons who stutter may gain increased fluency if shifting to a less automatic mode of speech, such as imitating an accent. This is despite that less automatized behaviors require more cerebral energy, at least at the cortical level (Schneider, 2009). Also the “adaptation effect,” i.e., that stuttering tends to be reduced by repeated reading of the same text, appears to be at odds with the energy deficiency hypothesis. The preliminary conclusion here is that these later arguments make it less likely that stuttering is the result of difficulties in sustaining the neuronal firing rate required for speech.

#### Proposal 2: Metabolic Limitations Result in Elevated Tonic Dopamine?

Anomalies of the dopamine system have been implicated in stuttering, in particular, because of pharmacological effects and theoretical links between stuttering and the basal ganglia (e.g.,Wu et al., 1997; Maguire et al., 2002; Alm, 2004; Chang and Guenther, 2020). In particular, a hyper-dopaminergic state has been proposed (Wu et al., 1997; Maguire et al., 2002), but also the existence of subgroups with hypo- and hyper-dopaminergic characteristics (Alm, 2004). An overview of the normal functions of the dopamine system is presented in Alm (unpublished manuscript).

An observation of relevance in the current context is that acute hypoxia and ischemia result in an elevation of the tonic level of dopamine in the synapses. This appears to be the result of a combination of mechanism: (1) release of dopamine without synaptic firing; (2) inhibition of the synaptic reuptake of dopamine; and (3) upregulation of tyrosine hydroxylase (TH), the rate-limiting enzyme in dopamine synthesis (Akiyama et al., 1991a,b; Norris and Millhorn, 1995; Gozal et al., 2005). I have not found studies of the effect of long–term mild limitations of the supply of energy on the tonic level of dopamine. If stuttering is related to a reduced supply of energy to the neurons, would this reduction be sufficient to result in a chronically elevated tonic level of dopamine? An elevated tonic level of dopamine will have effects on the dopaminergic signaling, causing an impaired signal-to-noise ratio, as the rapid phasic dopamine bursts will be smaller in relation to the baseline. Dopamine plays a central role in both automatization of speech movements and the execution of speech, in particular within the basal ganglia (Alm, unpublished manuscript). If metabolic impairments affect the dopamine system, it is quite possible that it is the changes in dopamine signaling that are the more proximal cause of stuttering. Based on this theoretical model it is essential to pursue the metabolic and the dopaminergic hypotheses of stuttering in parallel.

#### Are Metabolic Limitations Affecting the Cerebral Development of Speech?

At the group level, there appear to be some differences in the distribution of gray matter volume in children who stutter compared with other children. Recently, Chow et al. (2020) correlated this pattern with the normal expression pattern of lysosomal genes linked to stuttering. A correlation was found for two out of four genes. Similarly, Boley et al. (2021) correlated the pattern of gray matter differences with the normal pattern of cerebral glucose metabolism. A correlation of 0.36 was found for the left hemisphere. In both articles, it was argued that limitations in energy metabolism might affect childhood development, in particular during periods when energy utilization rapidly increased. A central aspect of these articles is the proposal that a limitation in metabolism during the childhood development of speech is linked to stuttering. This is in contrast to the hypotheses discussed previously in the present article, which have emphasized the acute effects of metabolic limitations. In conclusion, it is quite possible that a limitation in energy supply can have both developmental and momentary effects.

#### Peripheral and Central Dopamine Effects

##### Peripheral Dopamine

Dopamine also has functions outside the brain. Similar to other monoamines, dopamine can not pass through an intact blood-brain barrier (Hardebo and Owman, 1980). In relation to cerebral blood flow it is of interest that at low to moderate levels, blood dopamine has been shown to result in vasodilation and decreased systemic blood pressure, which contrasts with the effects of norepinephrine (Brodde, 1982; Reitsamer et al., 2004; Rubí and Maechler, 2010). Blood dopamine originates primarily from the sympathetic nervous system (Rubí and Maechler, 2010), which contains both dopaminergic and norepinephrinergic neurons (Bell, 1988). Stimuli that increase the sympathetic drive can have differential effects on the blood levels of dopamine and norepinephrine, with some stimuli resulting in an increase in both, while others elevate primarily one of them (Bell, 1988). In a study of five adults who stutter, the plasma levels of dopamine, norepinephrine, and epinephrine were analyzed (Rastatter and Harr, 1988). One participant showed a plasma dopamine level substantially above the reference level, whereas the other two catecholamines were within the normal range.

Homovanillic acid is the major metabolite of dopamine. The blood level of homovanillic acid is determined by a combination of cerebral and the peripheral dopamine metabolism (Amin et al., 1992). The blood level of homovanillic acid was studied in 92 children who stutter and in controls, aged 3 to 9 years, by Mohammadi et al. (2018). There was no statistically significant difference between the groups, and all the stuttering children were within the same range as the control children.

Overall, these results indicate that the level of peripheral dopamine is normal in most cases of stuttering.

##### Dopamine as a Regulator of Aerobic Glycolysis

There are interesting links between dopamine activity and cerebral aerobic glycolysis (i.e., consumption of glucose without consumption of oxygen when oxygen is available). Sato et al. (1991) found that intravenous injections of dopamine in cats treated with barbiturates resulted in a marked increase of cerebral glucose consumption while the oxygen consumption was reduced, i.e., there was increased aerobic glycolysis. The dopamine injections also resulted in increased CBF and arterial blood pressure. Increased aerobic glycolysis caused by dopamine is supported by results from a study in mice (Leonard, 1975), in which the dopamine was injected into the ventricles.

In line with these results, DiNuzzo et al. (2015) proposed that the entire central monoamine system, comprising dopamine, norepinephrine, serotonin, and histamine, modulates both the functioning and the metabolism of cerebral regions by modulating glycogen mobilization in the astrocytes. According to Papadopoulos and Parnavelas (1991), the monoamine system provides dense innervation of norepinephrine and serotonin to every cortical region. In contrast, the cortical dopamine projections are more restricted, with a clear preference for motor regions, and there are stronger projections to multimodal sensory regions as compared with primary sensory regions (Papadopoulos and Parnavelas, 1991). Gaspar et al. (1989) reported that the strongest dopamine projections to the human cortex target the primary motor cortex, the premotor cortex, the SMA, the anterior cingulate cortex, and the insula, with lower densities in prefrontal regions, such as BA 9. These descriptions of the cortical distribution of dopamine do not exactly match the GI map in Figure 1, but they do so well enough to suggest a possible relationship. For example, in the temporal and the parietal lobes, the primary areas show lower GI than the multimodal parts, which is in parallel with the distribution of dopamine according to the review by Papadopoulos and Parnavelas (1991). The inner arch of the anterior cingulate cortex (i.e., BA24) shows low GI in Figure 1, which may be related to the phylogenetically older cortical structure there.

Another indication pointing towards a connection between GI and dopamine is the common decline of GI and dopamine with aging. The decline of GI in humans is illustrated in Figure 2. A substantial decline of cortical and subcortical dopamine with aging was reported by Goldman-Rakic and Brown (1981) in a study of rhesus monkeys. This was in contrast to the levels of norepinephrine and serotonin, which remained largely stable. In summary, it might be hypothesized that the distribution of dopaminergic projections to the neocortex contributes to the pattern of GI shown in Figure 1.

Recently, Maguire et al. (2021) discussed this possible interaction between dopamine and metabolism, with astrocytes as a mediating link. It has been shown that astrocytes carry both D1 and D2 receptors, and that pharmacological blockade of the D2 receptors increases the metabolic activity of astrocytes in rats (Konopaske et al., 2013). The study by Maguire et al. (2021), of persons who stutter, reported that the D2 antagonist risperidone resulted in increased uptake of glucose during speech, in the left striatum and in the Broca’s area. The stuttering was reduced in the risperidone-group, compared to the placebo group. The authors proposed that, in part, the effect of risperidone on stuttering involves an increase in metabolism by striatal astrocytes. This finding and hypothesis are in line with the hypothesis of ADHD suggested by Todd and Botteron (2001), as discussed above. Todd and Botteron hypothesized that dopamine affects the glycogen metabolism in astrocytes, in turn affecting the symptoms of ADHD. Overall, it seems that studies of the interactions between the metabolic system and the dopamine system may be of importance for the understanding of stuttering.

##### Dopamine, Aerobic Glycolysis, and Synaptic Plasticity

Synaptic plasticity in motor regions is directly related to the presence of dopamine in the cortex: it has been shown that cortical dopamine is required for long-term potentiation in skill and motor learning (Molina-Luna et al., 2009; Hosp and Luft, 2013). As discussed at page 3, it has been shown that sensorimotor learning results in temporarily increased aerobic glycolysis for hours after the training, for example in the BA44 (Shannon et al., 2016). This has been proposed to support the view that aerobic glycolysis is particularly involved in synaptic plasticity (Goyal et al., 2017). It is therefore possible that Figure 1 and Figure 2 reflect, at least partially, the momentary synaptic plasticity that is taking place in these participants. It is clear that, on average, a greater amount of learning and plasticity takes place at a young age. The mean age of the participants summarized in Figure 1 was 25 years. It is conceivable that the GI pattern changes over time with development, as different types of learning engage different regions. Considering the dynamic aspect of aerobic glycolysis, it would be of great interest to study persons at older ages after intense motor learning. Would they show a similar localized increase of aerobic glycolysis as is seen in younger persons? Furthermore, it would be of interest to study the aerobic glycolysis in cortical and subcortical regions of persons who stutter immediately after intense speech fluency training.

In summary, there are substantial indications linking cortical dopamine to energy metabolism, CBF, aerobic glycolysis, and synaptic plasticity. The functions of dopamine may be the factor that links the disparate observations reviewed in this article. How this might be linked to stuttering is still an open question.

### Can Diet Influence Stuttering?

Preliminary findings regarding the possible effect of thiamine on stuttering were discussed above. If the metabolic hypothesis is confirmed, are there other possible effects due to diet? It has long been recognized that a “ketogenic diet” may have a positive effect on epilepsy (see e.g., Schoeler et al., 2021). In a ketogenic diet, there is a large reduction in the intake of carbohydrates, and a shift to high-fat food, to induce the production of ketones. Lately, ketogenic diets have become relatively popular among the public, for weight loss. Ketones are produced by the liver from fatty acids. The exact mechanism by which a ketogenic diet has an effect on epilepsy is not clear, but ketone bodies alter the cerebral metabolism by bypassing glycolysis and increasing mitochondrial oxidation (Lutas and Yellen, 2013). In short, the ketone bodies produced by someone following a ketogenic diet provide an alternative metabolic pathway for neurons. If some instances of stuttering are related to an impairment of glycolysis, it would be of interest to explore the effect of a ketogenic diet. Discussion forums on the Internet provide some anecdotal reports of a reduction in stuttering due to a ketogenic diet. Of course, a ketogenic diet requires radical lifestyle changes that will limit its usefulness. Moreover, medical supervision is advised.

## Conclusions

This review points at several outstanding issues for the research on the nature of stuttering. Overall, it is clearly premature to draw conclusions regarding the pathophysiology of stuttering based on the data reviewed, and there is a need for further studies. Possibly, the strongest finding in this review was the consistent reports of the reduced power of the EEG beta band, in combination with higher homogeneity in the stuttering group for this measure, compared with the controls. It is currently difficult to explain the conflicting data for the absolute CBF. It can be noted that the pattern of the reported reduced rCBF in adults who stutter corresponds well with both the pattern for the glycolytic index and with the expression of the ARNT2 gene. The glycolytic index indicates the degree of reliance on aerobic glycolysis for normal brain function, whereas the ARNT2 gene is one part of the mechanism leading to induction of glycolysis. Alterations to the ARNT2 gene have been linked to stuttering.

Neocortical aerobic glycolysis appears to, at least partly, reflect the momentary processes of synaptic plasticity and learning. This makes it a dynamic process rather than a static property. Further, synaptic plasticity in the motor regions has been shown to require the presence of dopamine. It is here proposed that the spatial variations of the dopaminergic projections to the neocortex contribute to the pattern of GI shown in Figure 1.

The genes with the strongest established link to stuttering affect the transport of enzymes to the lysosomes, for degradation and recycling of biomolecules. It is of great interest that these gene variants do not appear to affect general health but result specifically in stuttering in humans and reduced vocalization in mice pups. It is likely that the lysosomal alterations result in a mild reduction in the processing rate of biomolecules. The link to energy metabolism is further strengthened by the observation that mice carrying these genes tended to have fewer astrocytes in their cerebral white matter.

The single report of extremely high blood levels of nitric oxide in children who stutter needs to be replicated. Nitric oxide is involved in the regulation of cerebral metabolism and blood flow. Therefore, it is of theoretical interest in this context. If the finding can be confirmed it would be of great importance to explore this further.

The hypothesis that stuttering may be related to a limitation of the supply of energy that is required to sustain rapid neuronal firing during speech was discussed. Even though this hypothesis is in line with several observations, it does not account for the fact that stuttering often occurs at the beginning of utterances, or that a shift to a less automatized mode of speaking (e.g., imitation of an accent) tends to have a fluency-inducing effect.

Another possibility discussed is that mild limitations in energy metabolism result in an elevated tonic level of synaptic dopamine, as demonstrated in acute hypoxia. The links between dopamine and energy metabolism indicate that the dopaminergic and the metabolic hypotheses of stuttering need to be explored in parallel.

## Data Availability Statement

The original contributions presented in the study are included in the article/Supplementary Material, further inquiries can be directed to the corresponding author.

## Author Contributions

PA is the sole author. The author confirms being the sole contributor of this work and has approved it for publication.

## Conflict of Interest

The author declares that the research was conducted in the absence of any commercial or financial relationships that could be construed as a potential conflict of interest.

## Publisher’s Note

All claims expressed in this article are solely those of the authors and do not necessarily represent those of their affiliated organizations, or those of the publisher, the editors and the reviewers. Any product that may be evaluated in this article, or claim that may be made by its manufacturer, is not guaranteed or endorsed by the publisher.

## Footnotes

^1^It may be mentioned that a problem when trying to capture the literature is that key terms such as glycolysis and aerobic appear to have been used with somewhat different meanings by different authors.

^2^Data from Jenson et al. (2020) were from passive exposure to auditory noise, so this was not fully from rest.

^3^In the article by Bilal et al. (2017), the interquartile range was presented instead of standard deviations. For the calculation of Cohen’s *d* effect size, the standard deviations were estimated based on the method from Higgins and Green (2011).

## Abbreviations

BA: Brodmann area
CBF: cerebral blood flow
EEG: electroencephalography
GI: glycolytic index
rCBF: regional cerebral blood flow
SMA: supplemental motor area
SNc: substantia nigra pars compacta.
